# Development of a coarse-grained model for surface-functionalized gold nanoparticles: towards an accurate description of their aggregation behavior†

**DOI:** 10.1039/d3sm00094j

**Published:** 2023-04-17

**Authors:** Emanuele Petretto, Pablo Campomanes, Stefano Vanni

**Affiliations:** a Department of Biology, University of Fribourg, Chemin du Musée 10 1700 Fribourg Switzerland stefano.vanni@unifr.ch

## Abstract

Understanding the dispersion stability and aggregation propensity of self-assembled monolayer gold NPs at a molecular level is crucial to guide their rational design and to inform about the optimal surface functionalization for specific applications. To reach this goal, *in silico* modeling *via* coarse-grained (CG) molecular dynamics (MD) simulations is a fundamental tool to complement the information acquired from experimental studies since CG modeling allows to get a deep knowledge of the molecular interactions that take place at the nanoscale in this kind of systems. Unfortunately, current CG models of monolayer-protected AuNPs present several drawbacks that limit their accuracy in certain scenarios. We here develop a CG model that is fully compatible and extends the SPICA/SDK (Shinoda–DeVane–Klein) force field. Our model allows reproducing the behavior of AuNPs functionalized with hydrophobic as well as charged and more hydrophilic ligands. This model improves upon results obtained with previously derived CG force fields and successfully describes NPs aggregation and self-assembly in aqueous solution.

## Abstract 2

Understanding the aggregation propensity of protected gold nanoparticles (NP) is crucial to guide their ligand shell rational design. We developed and validated an NP model for MD simulations capable of accurately describing aggregation.

## Graphical abstract

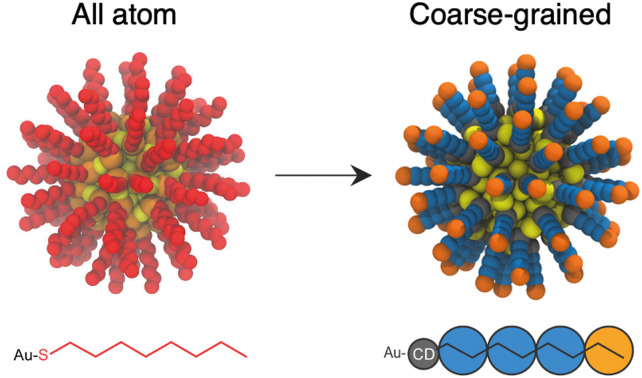

## 1. Introduction

Metal nanoparticles (NPs), particularly gold NPs, have drawn considerable interest due to their vast potential applications in numerous fields.^1,2^ These applications depend intimately on the NP's design: NPs must be active yet stable in the final solvent. Among other nano-structures, self-assembled monolayer gold NPs (SAM-AuNPs) are intensively investigated due to their extremely promising application in a variety of fields, including biology,^3^ sensing,^4^ and catalysis.^5^ In this kind of NPs, aliphatic molecules bind and protect the metal core *via* thiol–gold bonding. This external organic layer modulates the overall behavior of the NPs, from their specific chemical activity to their solvent media dispersion stability, and therefore defines SAM-AuNPs aggregation propensity. Changes in the organic layer composition by modifying the length of the ligands or their terminal functional groups allow to tailor NP's properties.

Although colloidal stability has been extensively experimentally studied, molecular interactions occurring at the nanoscale are still not entirely understood.^6–12^ Hence theoretical methods are nowadays employed to obtain complementary information and a more refined and essential understanding of NP aggregation. To develop accurate models able to predict NPs behavior, it is fundamental to adequately take into account both the interaction between NPs and their dynamics. Traditional continuum theories, such as the classical colloid science, Derjaguin–Landau–Verwey–Overbeek (DLVO) theory, have been used since a long time to estimate interaction energies between particles; however, these theories are not valid at short inter-particle distances because they fail to capture solvent polarization,^13^ hydration effects,^14^ and NP surface phenomena,^15^ and inadequately describe discrete size effects when the distance between particles is shorter than 2 nm.^16^ Moreover, as previously shown for both colloidal suspensions^17,18^ and polyelectrolyte solutions,^19,20^ the standard DLVO potential lacks a long-range attraction term. Consequently, DLVO-like theories fail to explain long-range attraction between similarly charged particles and unavoidably lead to inaccurate predictions in this kind of systems. For these reasons, computational techniques are commonly adopted to gain knowledge on inter-particle potentials and their connection with NPs aggregation. In particular, both the diffusion- and the reaction-limited cluster aggregation kinetics have been explored *via* Monte Carlo (MC) simulations.^21–25^ Nevertheless, the accuracy of these MC-based strategies is limited because, due to their reliance on simple sticking probability rules to simulate particle dynamics, they disregard thermal motion effects on aggregates detachment and rearrangement. In addition, they commonly use DLVO-like theories to compute the frequency of collisions between particles with all the drawbacks mentioned above.

Robust computational alternatives are molecular dynamics (MD) simulations. For example, all-atom (AA) simulations have been used to investigate NPs aggregation and concluded that this phenomenon is driven by short-range attraction with a potential energy characterized by a deep attractive well that stabilizes the dimer state.^26,27^ While most AA *in silico* experiments have so far focused on the study at the molecular level of NP–NP interactions in relatively small systems,^26–28^ coarse-grained (CG) simulations—because of their lower computational cost—appear as the method of choice for studying NPs self-assembly, as well as structure, kinetics, and dynamics of NPs aggregates.^29–31^

In this paper, we develop a CG model, which is fully compatible and extends the SPICA/SDK (Shinoda–DeVane–Klein) force field,^32–34^ to describe NPs aggregation and self-assembly in aqueous solutions. SPICA has been parameterized to match experimental properties such as surface/interfacial tension and density, and it has been shown to reproduce different properties at the interface between water and lipids with good accuracy.^33,35,36^ It also appears to be well adapted to describe phenomena in which surfactant-like molecules are involved.^34^ Nevertheless, although SPICA is a promising force field to describe the behavior of SAM-AuNPs in aqueous solution, it has an important disadvantage that limits its applicability range: the relatively reduced number of bead types that are currently available in this force field to map atomistic structures to their CG representation. In particular, a bead able to mimic the physicochemical properties of the grafting points (S atoms) at the surface of the NPs core is currently missing. In this work, we thus decided to extend the SPICA force field by incorporating a new bead, core-decoy (CD), to overcome this drawback and properly represent the NP's core. The purpose of including CD beads to describe the core of AuNPs is two-fold: (1) to properly describe the core-solvent affinity and (2) to serve as ligand-shell grafting-point locations. Here we show that our CG model can accurately mimic the NP–NP potential energy computed from atomistic MD simulations performed with the OPLS force field^37^ and that, among all the interactions, those between the NP's core and the solvent are crucial to reproduce NP–NP interaction energies. We then use this validated model to explore the dynamics and kinetics of aggregation for NPs containing both hydrophobic and hydrophilic ligands.

## 2. Materials and methods

### 2.1 Molecular dynamics simulations

#### 2.1.1 Unbiased AA simulations

**Fig. 1:**
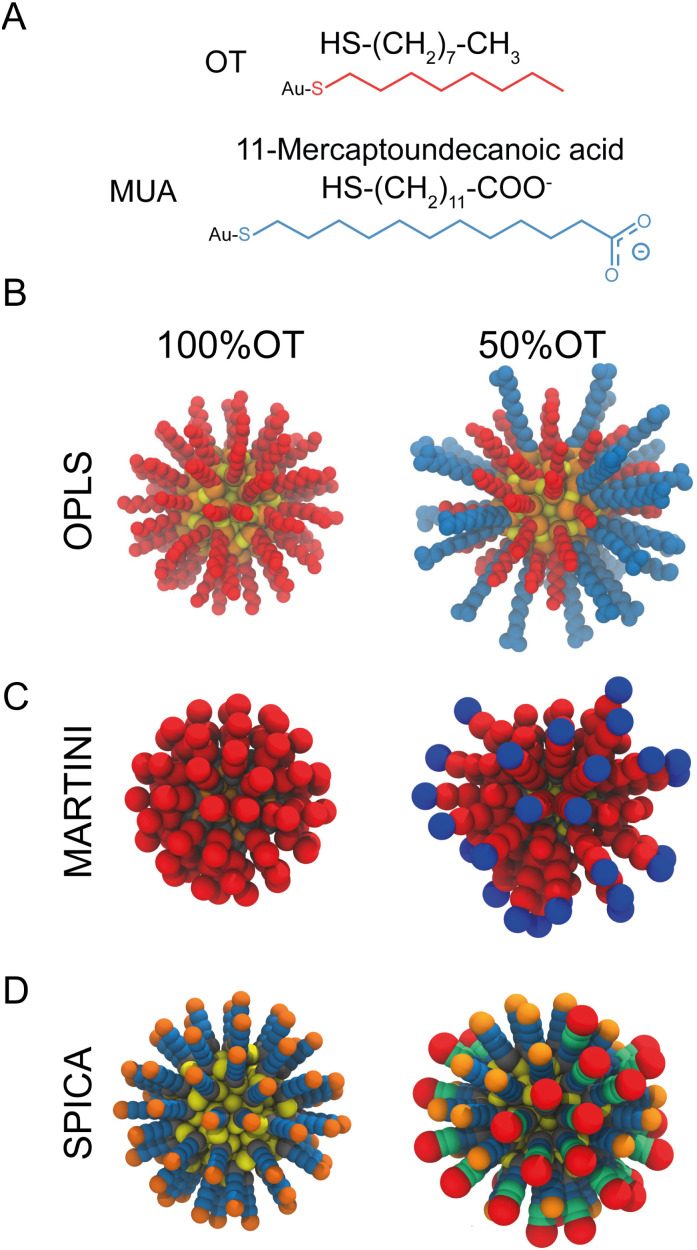
Structural and geometrical characteristics of the simulated NPs. (A) The surfactants forming the ligands shell; (B) NP models for 100%OT and 50%OT OPLS. To highlight the ligand shell arrangement, the ligands are represented with the same color code reported in panel A, OT (red), and MUA (blue); (C) NP models for 100%OT and 50%OT MARTINI. Here the ligands are represented to show the different beads used: two C1 beads (red) for OT, and two C1 beads (red), and a Qda bead (blue) for MUA;^52^ (D) NP models for 100%OT and 50%OT SPICA. Here, OT is composed of three CM2 beads (blue) and a CT2 (orange), and MUA is composed of two CM2 beads (blue), two CM beads (green), and an Asp bead (red).^33,34^

The atomistic simulations of the solvated mercapto-undecane carboxylic acid (MUA) and octanethiol (OT) functionalized Au-NPs were performed using a model comprising 144 Au atoms and 60 S atoms for the NP core. These sulfur atoms were then used as grafting points to 60 ligands, bound to the NP core *via* Au–S bonds. In particular, we designed NPs with two different ligand shell ratios: 100%OT and 50%OT : 50%MUA (“50%OT”) (Fig. 1A and B). The geometrical disposition of the grafted molecules is random. Parameters compatible with the OPLS^37^ forcefield, as derived by Salassi *et al.*,^38^ were used for the hydrophobic NPs, while the TIP3P model^39^ was employed for the water molecules.

All the MD simulations were run using the GROMACS v2020.x packages. The van der Waals (vdW) interactions were truncated using a cut-off value of 1.4 nm, and a switching function was applied to the tail region (1.2–1.4 nm) to smoothly bring the vdW potential to zero at the cut-off distance. The bonds involving hydrogen atoms were constrained using the LINCS^40^ and SETTLE^41^ algorithms. Electrostatic interactions were taken into account by means of the Particle Mesh Ewald (PME)^42^ algorithm (Fourier grid space of 0.12 nm and a 1.4 nm real-space cut-off).

To generate the configurations required for the parameterization, we simulated the dynamics of one NP in solution. This system was equilibrated using a Berendsen thermostat and barostat^43^ with coupling time constants of 2 ps (at 298 K and 1 atm). After initial equilibration of the system for 1 ns, we used a Nosé–Hoover thermostat^44^ and a Parrinello–Rahman barostat^45^ (at 298 K and 1 atm) with coupling time constants of 1 ps during production. These simulations were run for 50 ns to get enough statistics and well-converged values.

#### 2.1.2 Umbrella sampling (US) AA simulations

To generate the configurations required for the US simulations,^46^ we pulled two identical NPs away from an initial dimer state. NP-dimers were solvated in water and simulated in an orthogonal box with periodic boundary conditions. Box sizes of approximately 16 × 8 × 8 nm^3^ were used for both 50% OT and 100% OT dimer simulations. In the case of negatively charged NPs (50% OT), the system was neutralized by adding 30 Na^+^ per NP. The final ionic concentration (100 mM) was obtained by including an appropriate number of solvent molecules. The total number of particles in the simulation was of about ∼123 000. After a short equilibration, the NPs were separated with a force of 1300 kJ mol^−1^ nm^−2^ and at constant velocity (0.001 nm ps^−1^). From this pulling trajectory, we extracted configurations at equally spaced values (every 0.2 nm) along the selected reaction coordinate: the distance between the center of mass of the two NPs-core. For each umbrella window, the initial configuration was equilibrated (at 298 K and 1 atm) using a Berendsen thermostat and barostat^43^ with coupling time constants of 2 ps, while the interparticle distance was constrained at its original value. Afterwards, a 60 ns production run was carried out for every window. During production, a Nosé–Hoover thermostat^47^ and a Parrinello–Rahman barostat^45^ (with coupling time constants of 1 ps) were used to control the temperature (298 K) and pressure (1 atm), respectively. The potential of mean force (PMF) along the aforementioned reaction coordinate was reconstructed by using the weighted histogram analysis method (WHAM) in order to combine the umbrella histograms obtained for each window.^44^

### 2.2 SPICA CG simulations

All the MD simulations with the SPICA force field^32–34,36,48^ were performed using the LAMMPS package.^49^ Only a new bead (CD) had to be introduced to account for and mimic the physico-chemical properties of the grafting points at the surface of the gold core. All the other CG parameters were adopted from previous studies.^32–34,36,48^ To generate the configurations required for the parameterization, we performed unbiased CG simulations of isolated 100%OT NPs in solution using various solvents (hexane, heptane, octane, and nonane) that collectively contain all the beads present in the SPICA coarse-grained mapping for OT (CM2, CM, CT2) (Fig. 1D). Orthogonal boxes of approximately 10 × 10 × 10 nm^3^, thus amounting up to ∼20 000 particles, were used for the simulations, which were performed using periodic boundary conditions. In addition, we also performed simulations of the NPs in water solution to obtain reliable parameters for the CD : W interaction. For each of the solvents mentioned above, an initial equilibration run (10 ns) was followed by a production simulation that was extended until convergence (about 37 ns). All the runs were executed in the NPT ensemble using a Nosé–Hoover thermostat^44^ and barostat^50^ to control the temperature (298 K) and pressure (1 atm), respectively. We employed an integration time step of 15 fs. The NPs core was considered as a unique rigid body using the fixed rigid implementation in LAMMPS. Nonbonded interactions were truncated using a 15 Å cut-off, whereas electrostatic interactions were taken into account by means of the PME algorithm.^42^

To investigate multi-NPs aggregation, twenty-seven NPs (arranged in 3 × 3 × 3 nm^3^, 8.3 nm apart) were placed in an orthogonal box with a size of approximately 25 × 25 × 25 nm^3^. The total number of particles in the simulation was approximately 180 000. The systems (composed by either 100%OT or 50%OT in water solution) were equilibrated for 60 ns and, after that, simulated for 1000 ns. Three replicas were run for every system to get enough statistics for subsequent analyses.

To validate our SPICA-compatible model, we also estimated the PMF profile corresponding to NPs dimerization using US. These simulations were performed using MD parameters and methodologies analogous to those employed for the unbiased runs and a protocol and US parameters identical to those described in the previous section (US AA simulations). The solvated dimeric systems contained approximately 40 000 particles.

### 2.3 MARTINI CG simulations

All MARTINI simulations were performed using the GROMACS v2020.x package^51^ and the MARTINI2 force field^52^ (Fig. 1C). van der Waals interactions were truncated at a cut-off distance of 1.1 nm, and the Verlet cut-off scheme was used for the potential shift. Coulombic terms were calculated using the reaction field method with a cut-off distance of 1.1 nm. Production runs were performed at 298 K using a velocity-rescale thermostat,^53^ a Parrinello Rahman barostat,^45^ and an integration time step of 20 fs. As done for SPICA, in the case of MARTINI, we also estimated the PMF for NPs dimerization along the selected reaction coordinate using US. The pulling simulations, sampling per umbrella window, and subsequent analyses were carried out using protocols, techniques, and parameters analogous to those described above for the AA simulations. The total number of particles in these simulations was of ∼31 000.

In addition, we also investigated multi-NPs aggregation by means of unbiased simulations. To this end, twenty-seven NPs (arranged in 3 × 3 × 3 nm^3^, 8.3 nm apart) were placed in an orthogonal box with a size of approximately 25 × 25 × 25 nm^3^. This system, which contained about 130 000 particles, was equilibrated for 60 ns. Subsequently, three independent replicas were run for 1000 ns each. Both equilibration and production were carried out using a Nosé–Hoover thermostat and barostat^47^ to control the temperature and the pressure (298 K and 1 atm, respectively). The convergence of these simulations was assessed by computing the variation in the formation of new NP aggregates with time, fitting this variation to an exponential curve, and verifying that the extrapolation from 1 μs to a final time equal to 10 μs led to a negligible variation (smaller that 0.01).

### 2.4 CG model

The SPICA CG model uses several simple interaction functions to describe molecules. Harmonic bonds stretching (for 1–2 bonded pairs) and angle bending potential (for 1–2–3 bonded pairs) are employed for intramolecular interactions. Pairs separated by more than two bonds interact *via* nonbonded forces.1
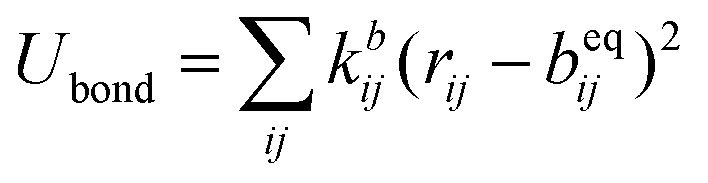
2
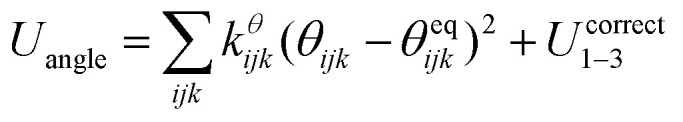
3
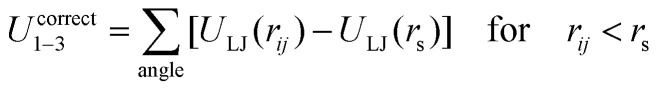
4
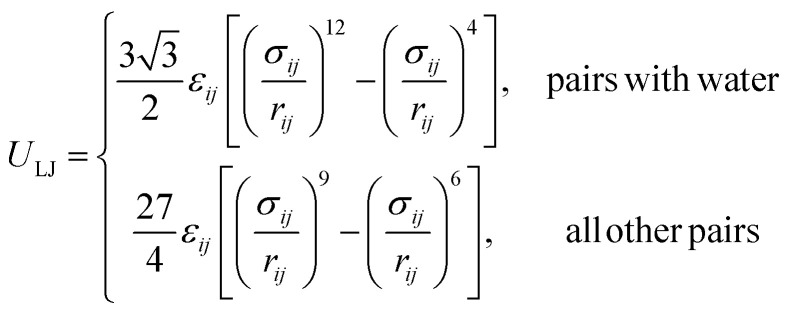
*k*^*b*^_*ij*_ and *k*^*θ*^_*ijk*_ are the force constants, and *b*^eq^_*ij*_ and *θ*^eq^_*ijk*_ are the distances and angles that correspond to minimum energy configurations, respectively. On top of the angle bending potential, a correction term for 1–3 interactions can be added to prevent angle collapses when small force constants are employed. Regarding the nonbonded interactions, the interactions between neutral beads are described using Lennard-Jones (LJ) potentials: a 9-6 LJ functional form is used for interactions between beads, except for those involving water for which a 12-4 LJ functional form is employed.

### 2.5 Derivation of CG parameters

#### 2.5.1 NPs core

For the CG representation of the NPs core, we employ a 1 : 1 mapping scheme. According to this scheme, in analogy to the OPLS model, each NP core atom is defined by a SPICA bead. Moreover, our CG model globally treats all gold beads and S grafting points as a rigid body system, and the LJ interactions between Au and any other bead as well as those between S beads are fully repulsive. According to this approach, all interactions involving the NP core are collectively defined and driven uniquely by the S beads, which warrants the investigation of large NPs without having to explicitly consider all the internal degrees of freedom of the NPs core. For this reason, the S beads are called ‘core-decoy’ beads (CD).

#### 2.5.2 Core-decoy beads

We developed parameters for all bonded interactions concerning the new bead (CD). To this end, the equilibrium values and force constants of all bonds and angles in which CD was involved were adjusted to reproduce the distance and angular distributions obtained in atomistic simulations. Using this procedure, we specifically parameterized: (1) the bond between the grafting point, CD, and the nearest bead, A, (CD–A); and (2) the angle formed between these two beads and the bead directly connected to A (∠CD–A–B). To derive all nonbonded LJ parameters (*σ* and *ε*) required to describe the interactions involving CD, we used a three-step protocol: (1) given two sets of *σ* and *ε* values, which define the space to explore, we generated an interactions matrix composed by all the possible *σ*/*ε* pairs. For each of these pairs, we run a short CG simulation of a single solvated NP and, from this simulation, we collected two different features: the radial distribution function (RDF) of the solvent and that of the ligand shell relative to the center of mass of the NP core; (2) the CG distributions were then compared with target AA data using relative entropy as metric:5
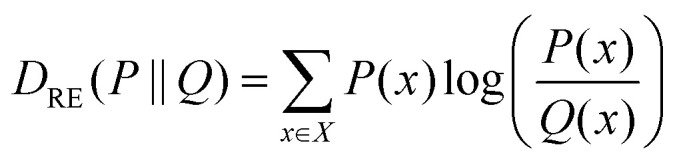
where *P*(*x*) and *Q*(*x*) are the probability distributions for the CG and target AA features, respectively. This allows to build a relative entropy surface for every feature using the *σ*/*ε* values as independent variables. According to the definition above, the smaller the relative entropy value the better the model superposition; (3) after a fitting and scoring procedure, these surfaces were summed up to generate a unique discretized matrix. The smallest values of this matrix correspond to the most suitable *σ*/*ε* pairs. In detail, each of the relative entropy surfaces was fitted to a multivariate polynomial regression model. To choose the appropriate polynomial order, the data was divided into a training set and a test set (using an 80 : 20 ratio). The data in the training set was used to build different models of increasing polynomial degree, and then the model which minimized the mean squared error on the test set was selected. Subsequently, the surfaces were resampled to increase their resolution, normalized by the number of elements, and summed to find the intersection between them. The intersecting points represent the best possible *σ* and *ε* non-diagonal parameters for both the explored features. To avoid numerical artifacts, we selected the best *σ*/*ε* pair after convoluting the surface with a kernel that averages out the first Euclidean neighbours of every element.

#### 2.5.3 Au–Au and CD–CD interactions

Au–Au interactions. The LJ parameters for Au–Au interactions are fully repulsive and set as *σ*_Au–Au_ = 4 Å, *ε*_Au–Au_ = 0.0001 kJ mol^−1^. The initial educated guess for *σ*_Au–Au_ was 3.3 Å,^54^ but due to the core rigidity and to avoid solvent instabilities, such as freezing or permeation inside the NP core, we had to increase the *σ* value for this interaction. Au beads have no charge.

CD–CD interactions. The LJ parameters for CD–CD interactions are fully repulsive and set as *σ*_CD–CD_ = 2.785 Å, *ε*_Au–Au_ = 0.0001 kJ mol^−1^. CD beads are electrostatically neutral.

## 3. Results

### 3.1 Derivation of a coarse-grained model for surface functionalized Au-NPs

To properly describe the behavior of SAM-AuNPs in aqueous solution *via* CG simulations, it is imperative to have a CG model able to reproduce both NP–NP and NP–solvent interactions. For this CG model to be reliable, it is important to design an adequate AA to CG mapping and develop accurate bonded and nonbonded parameters. Both octanethiol (OT) and 11-mercaptoundecanoic acid (MUA) ligands can be mapped to a CG representation using beads already existent in the SPICA force field.^33^ However, as mentioned above, a new bead (CD), fully compatible with those currently existent in SPICA, had to be introduced to mimic the physicochemical properties of the NPs core surface with enough accuracy.

Regarding the SPICA force field development, the typical workflow to derive nonbonded parameters for new molecules is based on a top-down approach, by targeting macroscopic properties such as density and surface tension,^33^ while, on the other hand, parameters to describe the bonded interactions are commonly obtained through iterative Boltzmann inversion; *i.e.*, *via* an iterative procedure that leads to a consistent fit between the bond and angular probability distributions coming from AA and CG simulations. Notably, in this work, because of the lack of appropriate hands-on experimental data on NPs, we partially deviated from this procedure and also determined optimal nonbonded LJ parameters using results from atomistic simulations. In particular, since we focused here on the simulation of hydrophobic OT AuNPs (100%OT NPs) and charged and more hydrophilic OT/MUA (1 : 1 ratio) AuNPs (50%OT NPs), we had to derive all bonded and non-diagonal LJ parameters (*σ*_*ij*_, *ε*_*ij*_) required to describe the interactions between any of the beads composing these two ligands and CD. Moreover, we had also to develop parameters able to characterize the interaction of CD with water to study the behaviour of this kind of NPs in aqueous solution.

**Fig. 2:**
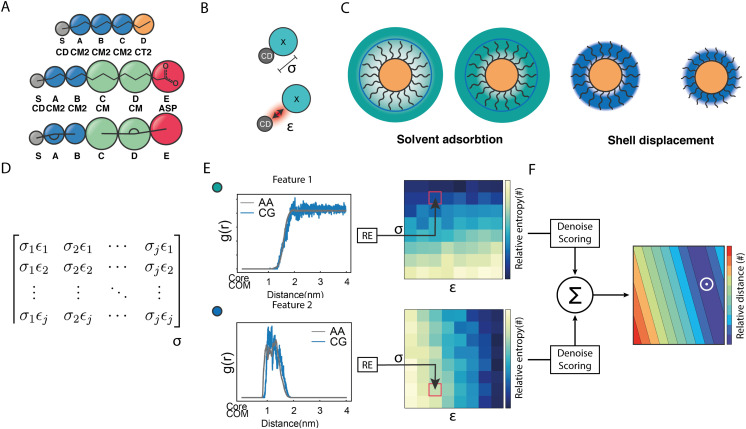
Protocol to build the coarse-grained model for NPs. (A) CG mappings for OT and MUA ligands (including the grafting point, S). (B) graphical interpretation of CD *σ* and *ε* non-diagonal parameters. (C) core–solvent effects arising from the solvent absorption around the NP and the ligand shell displacement. (D) Interactions matrix, which contains all *σ* and *ε* pairs explored during the grid-search approach. (E) Selected properties: the RDF of the solvent around the NP and the RDF of the ligand shell. These features are then compared with those obtained in atomistic simulations using Relative Entropy, thus generating a relative entropy surface. (F) After the fitting and scoring procedure, these surfaces are summed up to build a unique discretized matrix. The smaller values of this matrix correspond to the most suitable (*σε*) pair (white circle).

As shown in Fig. 2A, besides the grafting bead (S), four (A–D) and five (A–E) beads were used to map the OT and the MUA ligands, respectively. To properly describe the interactions between this kind of NPs and the solvent, we functionally redefined the grafting point, S, on the NPs surface as a ‘core decoy’ bead (CD) (Fig. 2B). Then, CD beads have two main functions: (1) describing the core-solvent affinity; and (2) serving as ligand-shell grafting-point locations. Changing the core-solvent affinity alters the absorption of solvent molecules around the NP and displaces the ligand shell. These two effects are strictly correlated since the solvent's abundance around the NPs influences the displacement of the ligand shell (Fig. 2C).

Regarding the bonded interactions, the bond between the grafting point S and the first bead A (S–A) and the S–A–B angle (SAB) had to be parameterized. These interactions are present in both ligands. Moreover, the bond between D and E (D and E) and the C–D–E angle (CDE) were also parameterized in the case of MUA (Fig. 2A). We derived the bonded terms of the coarse-grained potential *via* Boltzmann inversion by using the bond and angular distributions obtained in an atomistic simulation of solvated 50%OT NPs. ESI,† Tables S1 and S3 show the optimal values.

Concerning the nonbonded terms, we developed *σ* and *ε* parameters for all the LJ interactions including the new bead (CD). To this end, we used a protocol based on a grid-search approach that allowed us to refine an initial educated guess for every unknown non-diagonal parameter describing the interaction of CD with any other bead. The *σ*_*ij*_ and *ε*_*ij*_ combinations used as input for the protocol were composed by pairs of *σ* and *ε* values in the vicinity of the educated guess, which was determined using the Lorentz–Berthelot combination rules.^38,55^ Then, once the space to be explored during refinement was chosen, we had to perform a total of *n* CG simulations, where *n* is the number of elements in the interactions matrix (Fig. 2D), to compute the following features: (1) the RDF of the solvent molecules around a given NP and (2) the RDF of the last bead in the ligand shell relative to the centre of mass of the NP core. We also estimated the same properties from atomistic simulations carried out in analogous systems. This allowed us to compare the RDFs obtained using both AA and CG representations. This comparison was carried out using relative entropy (RE) as metric (eqn (5)). After polynomial fitting, resampling, and normalization, the elements constituting the RE matrices were summed up to infer those *σ* and *ε* non-diagonal parameters able to simultaneously minimize the information contained in both RE matrices (Fig. 2F) (see Materials and methods for details). As discussed below, the selected RDFs allowed us to discriminate between different scenarios nicely and to estimate the LJ parameters able to properly describe the interactions within and between NPs as well as the interplay between them and their environment.

**Fig. 3:**
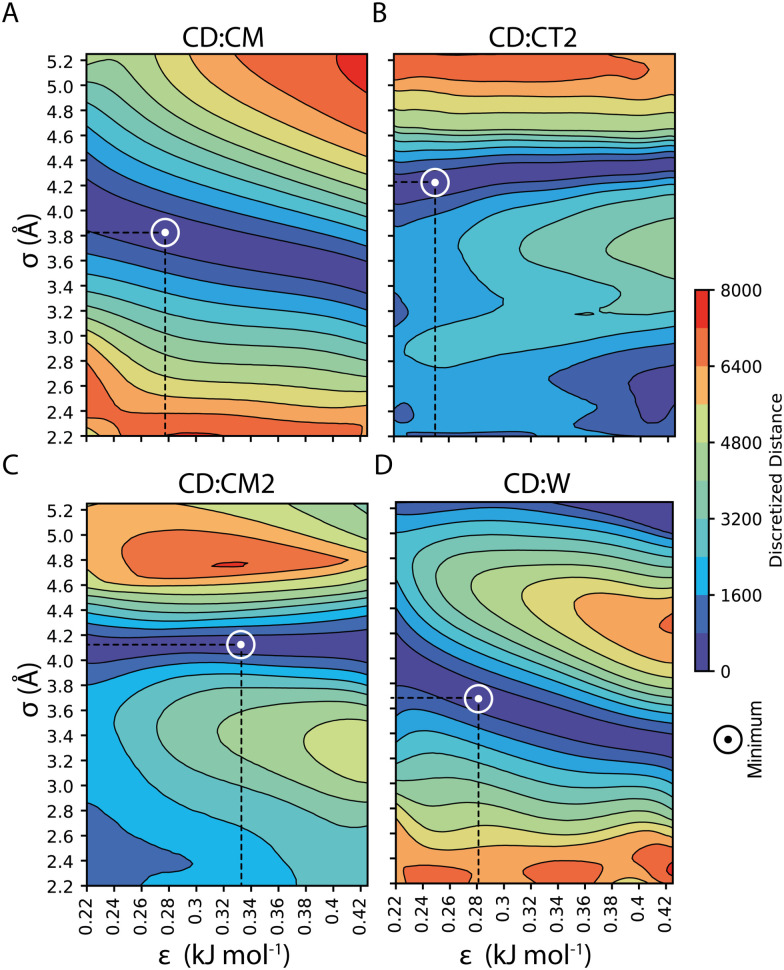
CD *σ* and *ε* optimal non-diagonal parameters: (A) CD : CM; (B) CD : CT2; (C) CD : CM2; and (D) CD : W.

Our parameterization strategy consisted of three consecutive steps. First, we developed LJ parameters to treat core–shell interactions in 100%OT NPs; then, we incorporated those required to define core–water interactions; finally, we extended the parameters set to the treatment of MUA-containing (50%OT) NPs. For the first step, we kept compatibility with the SPICA force field by selecting various small aliphatic molecules that fulfilled several criteria: (1) they were composed by already existent beads; (2) they collectively contained beads present in the OT and MUA coarse-grained mapping (CM, CT2, CM2); and (3) they could be employed as solvents in our simulations. With this in mind, we chose hexane, heptane, octane, and nonane as solvents and carried out MD simulations containing 100%OT NPs as solutes in each of these solvents. With this strategy, we aimed to jointly use information from the interactions of CD with the aliphatic solvents as well as with the aliphatic shell for the parameterization. As shown in Fig. 3, and Table 1, this choice allowed us to derive the *σ* and *ε* non-diagonal parameters corresponding to the following CD : shell interactions: CD : CM, CD : CT2, and CD : CM2. Although not strictly required to simulate the behavior of the selected 100%OT and 50%OT NPs in water solution, CD : CT interactions were also obtained as part of the parameterization pipeline (Fig. S1, panel A, ESI†) and are reported in ESI† Table S3 and Fig. S1. Then, using the optimized CD : shell values, we run simulations of 100%OT NPs in aqueous solution to get the optimal values for *σ*_CD–W_ and *ε*_CD–W_ (reported in Fig. 3 and Table 1) in an analogous way.

**Table 1:** CD *σ* and *ε* non-diagonal parameters optimization: CD : CM, CD : CT2, CD : CM2, CD : W, CD : SOD, and CD : CLA

Pair	*ε* (kJ mol^−1^)	*σ* (Å)
CD : CM	0.2760	3.798
CD : CT2	0.3284	4.078
CD : CM2	0.2489	4.176
CD : W	0.2517	3.774
CD : SOD	0.2517	3.774
CD : CLA	0.2517	3.774

### 3.2 Model validation

**Fig. 4:**
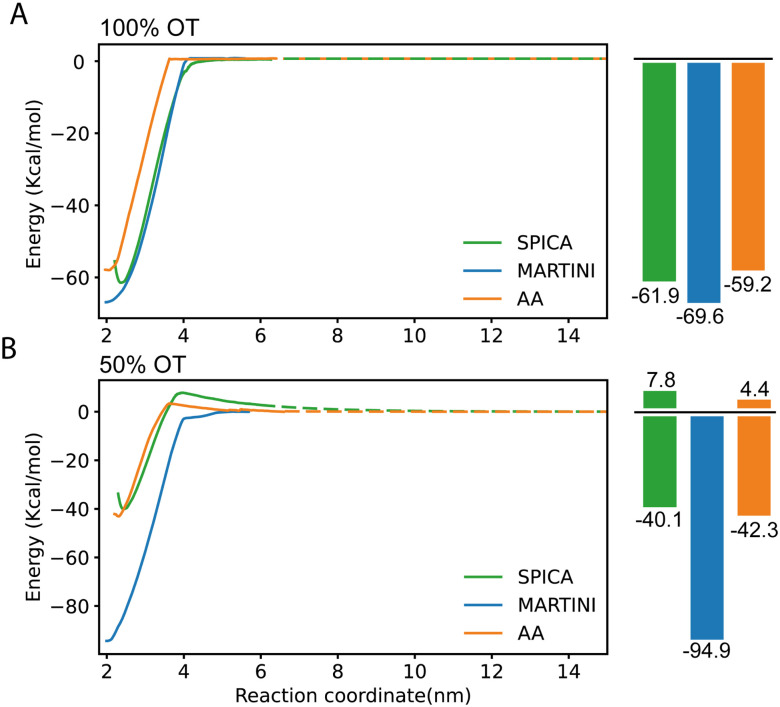
PMF profiles corresponding to the dimerization of SAM-NPs and estimated using SPICA, MARTINI, and OPLS (AA) force fields. (A) 100% OT NPs; (B) 50% OT NPs.

We next evaluated the ability of our protocol and the parameters here developed to reproduce free energies of dimerization between identical (either 100% or 50%OT) NPs. This process has been previously characterized using atomistic simulations, and the findings of these simulations experimentally supported by collecting tomographic EM images of the NPs in solution,^28^ thus providing a good validation test. As was the case for the AA simulations, we estimated the potential of mean force (PMF) for the dimerization process using the CG model developed in this work in combination with umbrella sampling (see Materials and methods for details). The profiles obtained are displayed in Fig. 4 and directly compared with those of AA simulations.

In the case of 100%OT NPs (Fig. 4A), the SPICA model, which incorporates the parameters developed here, presents a primary minimum that is 61.9 kcal mol^−1^ more stable than the fully separated state while a slightly lower stabilization (59.2 kcal mol^−1^) was reported in a previous study using AA simulations.^28^ Therefore, the new model successfully reproduces the free energy barrier found to separate the NPs from their dimer state, with only a small relative error of 4.36% with respect to the atomistic result. Notably, according to our CG model, the value along the selected reaction coordinate (distance between the center of mass of the NPs core) at which the interaction between the NPs results negligible (*i.e.*, the distance at which the free energy plateaus) is 3.9 nm, whereas this value is of 3.6 nm in the AA simulations. This implies that, when the CG model is used, the effective size of these NPs is 7.7% larger than the corresponding atomistic ones. The rationale for this size mismatch is due to the hydrophobicity of the 100%OT NPs and the size of the water beads; with the CG model, the first hydration shell is slightly displaced if compared to the AA one. We also computed the corresponding PMFs using the MARTINI2 force field.^52,56^ This force field has been successfully used to simulate numerous systems containing NPs to investigate NP–NP, NP–membrane, NP–protein interactions.^38,57,58^ As shown in (Fig. 4A), the MARTINI model overestimates the stability of the primary minimum (69.2 kJ mol^−1^) by 15.35% with respect to the atomistic result. Of interest, the SPICA and MARTINI force fields equally overestimate the distance at which NPs are no longer interacting.

To simulate the more complex MUA-containing (50%OT) NPs, we had to extend our CG model by choosing adequate LJ parameters for the CD : ASP interaction. In this case, because of the impossibility of using small molecules containing the ASP bead as solvents, we selected the parameters for the CD : ASP interaction by similarity. Since the CD : ASP interaction does not have an electrostatic character and because of the size similarity between ASP and CT2, we decided to use the *σ* and *ε* non-diagonal terms derived for CD : CT2 also for CD : ASP as a first approximation. Moreover, to perform simulations with charged NPs, we also had to select parameters to model the interaction between CD and the Na^+^ and Cl^−^ ions (SOD and CLA beads, respectively). To keep compatibility with the SPICA force field, we used the optimal CD : W LJ parameters to model the CD : SOD and CD : CLA interactions.^59^ The goodness of these choices was then investigated by assessing its ability to reproduce the PMF profile corresponding to the dimerization of 50%OT NPs.

For the charged 50%OT NPs, the SPICA and AA PMF profiles show a good agreement (Fig. 4B). In both cases, as expected because of the presence of charged ligands coating the NPs, the stability of the primary minimum with respect to the fully separated state is smaller than for 100%OT (40.1 kcal mol^−1^ and 42.3 kcal mol^−1^, respectively), and dimerization is not spontaneous anymore: both our CG model and the AA one display the presence of a free energy barrier (7.8 kcal mol^−1^ and 4.4 kcal mol^−1^, respectively) that must be overcome to reach the dimer state. Moreover, the barrier for NPs dimerization appears at a comparable interparticle distance. On the contrary, MARTINI leads to a PMF profile that differs even qualitatively. It clearly overestimates the stability of the primary minimum (94.9 kcal mol^−1^) with respect to that of the non-interacting NPs state and, furthermore, predicts the barrierless dimerization of the 50%OT NPs, making them virtually hydrophobic and much more attractive. Taken together, these results evidence that the CG model developed in this work can describe the dimerization of 100%OT and 50%OT NPs with a remarkable accuracy. On the other hand, MARTINI does a relatively good job to describe the behavior of the hydrophobic 100%OT NPs, but fails to reproduce the target PMF profile when the NPs present a more hydrophilic nature, such in the case of 50%OT NPs.

### 3.3 The aggregation of multiple NPs depends on the shell composition

**Fig. 5:**
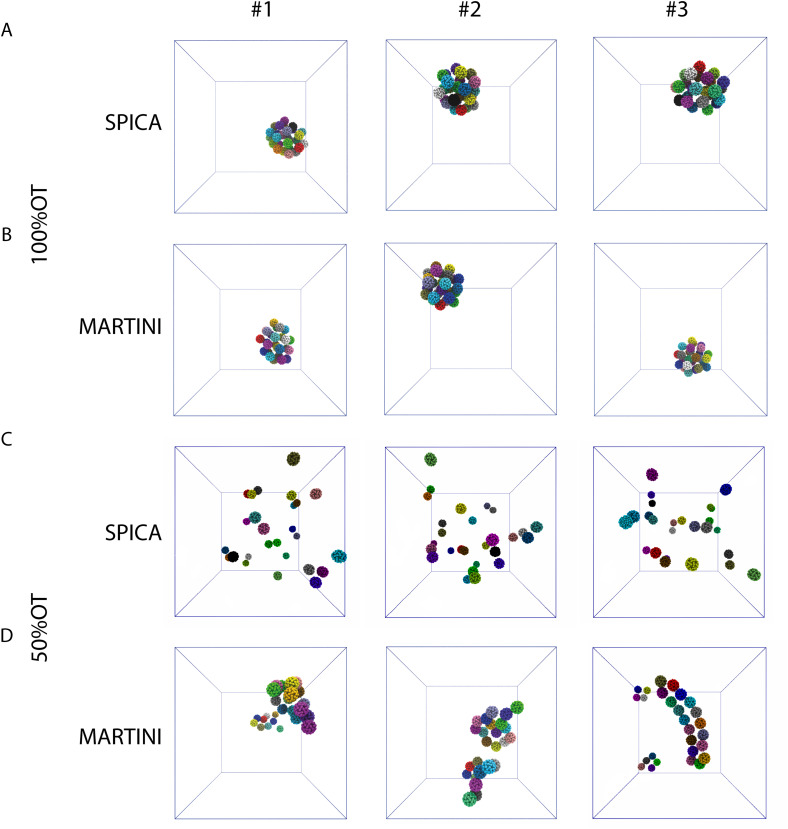
Structures representative of the final NP's aggregates (only NPs core is shown for clarity) found in the simulations: (A) 100%OT SPICA; (B) 100%OT MARTINI; (C) 50%OT SPICA; and (D) 50%OT MARTINI. The final structures of each of the three independent replicas run for every system and CG force field are displayed.

Next, we focused on the investigation of the aggregation of multiple 100%OT and 50%OT NPs in solution using our SPICA-compatible model, and we compared the results with those obtained with MARTINI. To this end, we placed 27 NPs in a 3 × 3 × 3 grid arrangement, solvated them with water, and added counterions to reach physiological conditions. Three replicas were run for each system. The results are displayed in Fig. 5.

The simulations performed with both CG models (SPICA and MARTINI) show that the 100%OT NPs aggregate within 1 μs of simulation. In both cases, these hydrophobic NPs show a high aggregation propensity. Indeed, all the NPs present in the system under study formed a unique compact cluster after short simulation times (a few hundreds of ns) (Fig. 5A, B and Fig. S2A, ESI†), and this morphology was maintained during the time scale of our simulations. On the other hand, 50%OT NPs simulated with SPICA and MARTINI force fields are significantly different from 100%OT and from each other too. When SPICA is used, the aggregates form dimers and trimers (Fig. 5C and Fig. S2B, ESI†); in contrast, MARTINI leads to double-chain or single-chain spiral aggregates (Fig. 5D and Fig. S2C, ESI†).

In order to understand the kinetics of the process, we computed the fraction of NPs partaking in a cluster; *i.e.*, we computed the fraction of NPs that were at least in a dimer state and not anymore free in solution. Fig. 5D reports the kinetics of aggregation, showing the percentage of aggregated NPs during the simulations for the three replicas. In general, no single NPs were found after 300 ns.

**Fig. 6:**
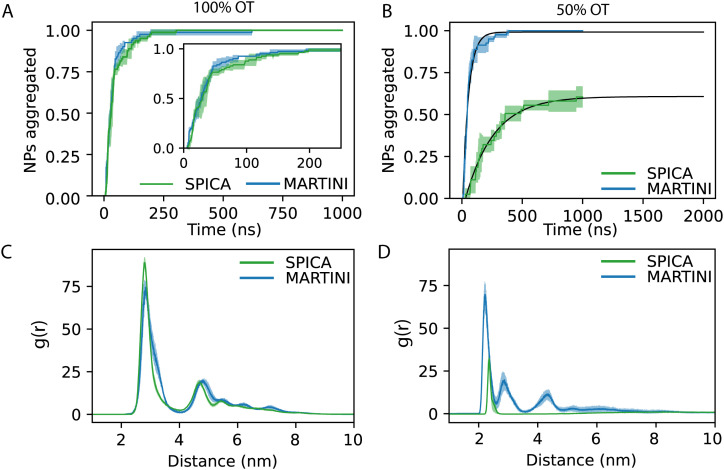
Aggregation behavior of 100%OT and 50%OT SPICA and MARTINI NPs: (A) kinetics of aggregation for 100%OT NPs; (B) kinetics of aggregation for 50%OT NPs; (C) RDF of 100%OT SPICA and MARTINI NPs; (D) RDF of 50%OT SPICA and MARTINI NPs.

From the aggregation's kinetics in 100%OT SPICA, it is evident that no single NPs are observed after the first ∼200 ns of simulation (Fig. 6A, green) and, with MARTINI (Fig. 6A, blue), the aggregation kinetics is remarkably similar to that found with SPICA, although appears slightly faster (Fig. 6A, inlet). Reasonably, this can be an effect of the larger number of clusters generated at the beginning of the simulation. Both CG force fields report the same overall structure for the final aggregates. Indeed, as shown in Fig. 6C, the radial distribution of the NPs is very similar once the simulations are converged.

On the other hand, as expected from the PMF profiles, 50%OT SPICA NPs are stable in solution. The aggregation kinetics shows that single NPs persist until 1 μs runtime (Fig. 6B). The first peak and shoulder of the RDF show the presence of dimers and triangular trimers(Fig. 6D). An exponential fit (*r*^2^ = 0.87) of the kinetic profile shows that only ∼60% of the NPs will aggregate, which stresses the stability of the NPs in solution. This result contrasts with the aggregation profile observed for 50%OT MARTINI NPs where, after ∼500 ns of simulation, there is no single NPs free in solution. In addition to kinetics, we also explored aggregation dynamics by studying the number of clusters and the number of NPs per cluster (Fig. S3, ESI†). From these analyses, two main conclusions arise: (1) cluster formation is generally faster for 100%OT NPs; (2) 50%OT MARTINI and SPICA NPs behave differently: in particular, 50%OT SPICA NPs result stable in solution, whereas 50%OT MARTINI NPs quickly form aggregates.

## 4. Conclusions

In this work, we developed a CG model able to accurately simulate the aggregation behavior of SAM-AuNPs in water solution. This model is fully compatible with the SPICA force field. To appropriately describe the solvent/NP interactions, we parameterized the ligand grafting point on NPs surface as a ‘core decoy’ (CD) bead. Due to the lack of hands-on experimental data on this kind of NPs, we partially deviated from the typical workflow used to incorporate new molecules in SPICA and determined all optimal nonbonded LJ parameters required to model the interactions in which the new bead, CD, was included using results from atomistic simulations.

In previous work, we have extensively characterized the dimerization profile of identical NPs. Therefore, with the new parameters obtained in this study, we first estimated the PMF dimerization profiles for both hydrophobic (100%OT) as well as charged and more hydrophilic (50%OT) NPs. We found that our SPICA-compatible CG model can adequately, still with some limitations, reproduce the results previously reported using AA simulations in both 100%OT and 50%OT NPs. We observed two main differences when comparing the NPs dimerization PMF profiles obtained using our new model and those predicted by the above-mentioned AA simulations: (1) the effective size of the 100%OT NPs is slightly larger in the CG model because of the displacement of the first hydration shell around the fully hydrophobic NPs coating; (2) in the case of 50%OT NPs, the dimerization barrier is slightly overestimated when using the CG model, and the electrostatic repulsion shows a longer exponential decay.

On the other hand, MARTINI fails to describe the behavior of the charged and more hydrophilic 50%OT NPs: it neglects the existence of the free energy barrier for the dimerization of this kind of NPs, and strongly overestimates the stability of the primary minimum, which corresponds to the dimer state. This can be partially due to the different schemes used to treat the long-range electrostatic interactions in both models: SPICA uses PME, whereas MARTINI uses a cut-off scheme.

Regarding the kinetics and dynamics of NPs aggregation, our model behaves as expected: (1) hydrophobic NPs in water inevitably attract each other and aggregate in the primary minimum (core-to-core) and (2) charged NPs are stable in water and form only dimers and trimers.^28^ Of note, the description of the ions itself is also a critical aspect of both CG force fields.^59–61^ The force fields represent ions as large-sized beads that describe the ion and its first hydration layer.^60^

Taken together, our results indicate that our SPICA-compatible model accurately describes the spontaneous aggregation of NPs in aqueous solution and allows to reliably mimic the physicochemical properties of NPs coated with different ligands. Therefore, although considerable work is still needed, it seems to be an accurate and good alternative to MARTINI for describing the behavior of NPs in a biological-like context,^2,62–64^ including the interactions between NPs and proteins or cellular membranes.^38,58,65^

In summary, here we developed a new CG model for NPs that is fully compatible with the SPICA force field and can accurately reproduce dimerization-free energy profiles obtained in previous studies *via* atomistic simulations. Using this model, we explored the aggregation kinetics and dynamics of both hydrophobic and hydrophilic/charged NPs. In addition, two secondary results arising from the parameterization protocol here employed are noteworthy: (1) in the absence of experimental data, the use of simple geometrical features such as the core-solvent and the core–shell RDFs seems to be a reliable alternative to get non-bonded parameters in ligand-coated NPs; (2) the interaction between the NPs core and the solvent is fundamental and must be adequately described to reproduce dimerization free energy profiles and, in particular, the characteristics of the primary minimum that defines the irreversible dimerization of this kind of NPs.

## Author contributions

E. P. and S. V. conceptualized and designed the research; E. P. performed the MD simulations and data analyses with the help of P. C.; S. V. supervised the project; the manuscript was written through the contributions of all authors.

## Conflicts of interest

There are no conflicts to declare.

## Supplementary Material

SM-019-D3SM00094J-s001

SM-019-D3SM00094J-s002
