# Supplementary material for: Development of a coarse-grained model for surface-functionalized gold nanoparticles: towards an accurate description of their aggregation behavior
Source: Soft Matter. 2023 Apr 17;19(18):3290–300. doi: 10.1039/d3sm00094j (PMC10170483; doi:10.1039/d3sm00094j)
Supplement: SM-019-D3SM00094J-s001 [file SM-019-D3SM00094J-s001.pdf]

Electronic Supplementary Material (ESI) for Soft Matter.
This journal is © The Royal Society of Chemistry 2023

Electronic Supplementary Information for:

Development of a coarse-grained model for surface-functionalized gold nanoparticles: Towards an accurate description of their aggregation behavior

Emanuele Petretto,[a] Pablo Campomanes,[a] and Stefano Vanni*[a]

[a] *Department of Biology, University of Fribourg, Chemin du Musée 10, 1700 Fribourg, Switzerland. E-mail: stefano.vanni@unifr.ch*

Supplementary Results

The dynamics of multiple NPs aggregation depends on their shell composition

To understand the dynamics of the aggregation between multiple NPs, we introduced the concept of geometrical cluster. We defined a geometrical cluster as a group of neighboring NPs which are at a given distance. A geometrical cluster, in general, does not describe a physical aggregate but it is merely used to investigate how close the NPs are in the simulation box. We evaluated geometrical clusters at increasing distances (from 2 to 13 nm). Figure S3 (panels A, C, E, G) shows, as heatmaps, the number of geometrical clusters that can be found at a given cut-off distance and as a function of time (rows) for each of the three replicas (columns). Among all possible distances, we further selected a specific subset that corresponds to the geometrical cluster at a 3.5 nm cut-off. This subset, in particular, was used to define physical aggregate. We then analyzed the number of NPs within each geometrical cluster Figure S3 (panels B, D, F, H) for this particular subset. The maps displayed in these panels allow to describe the aggregation process qualitatively as a function of time. As shown in Figure S3, the overall aggregation dynamics depends on the NP type and CG forcefield.

For 100%OT NPs, the heatmaps obtained with both force fields (SPICA and MARTINI) are qualitatively similar: the number of aggregates reaches its maximum in the early stage of the simulations (∼50 ns) (Figures S3A and S3C), mainly generating dimers and trimers until complete aggregation, which was found on average at ∼700 ns (Figures S3B and S3D). In addition, in this kind of NPs, geometric clusters can join together with high probability if their distance is between 4 and 6 nm. This is graphically represented in Figures S3A and S3C. There, each region of continuous color constitutes a distance interval characterized by the same number of geometrical clusters and, therefore, the highlighted circles at the right side of these regions serve as a qualitative assessment of the aforementioned regrouping.

Regarding the 50%OT NPs, the heatmaps corresponding to the geometrical clusters show an entirely different profile for SPICA and MARTINI. In the SPICA model, the maximum number of geometrical clusters (∼10) is reached in $\sim 1\mu$s (Figure S3E), and dimers and trimers persist until the end of simulation time (Figure S3F). In contrast, in the simulations with MARTINI, the maximum number of groups (∼8) is reached in ∼80 ns. Interestingly, the heatmaps corresponding to the geometrical clusters are similar to those obtained for the 100%OT NPs, but the NPs do not aggregate into a single group (Figures S3E and S3F) in any of the three replicas.

In summary, these analyses highlight that cluster formation is generally slower for 100%OT NPs, and that 50%OT MARTINI and 50%OT SPICA NPs exhibited disparate behaviors. Specifically, 50%OT SPICA NPs remained stable in the solution, whereas 50%OT MARTINI NPs rapidly formed aggregates.

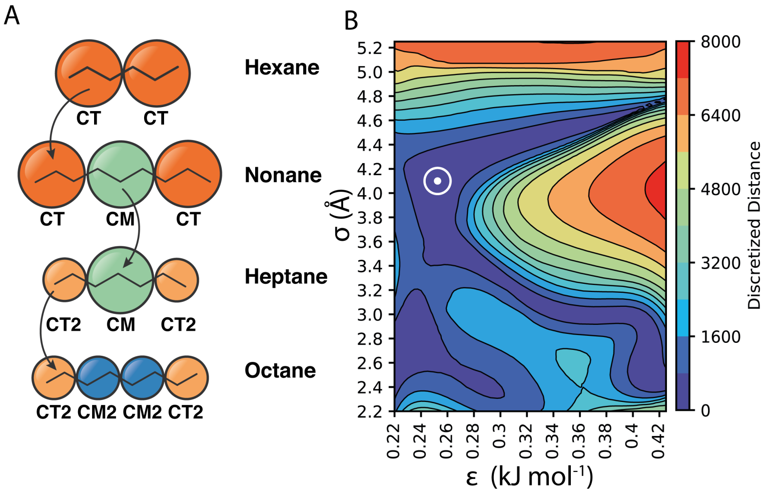

Figure S1: Pipeline followed to get optimal σ and ε non–diagonal parameters for CD. A) We started with hexane (CT–CT) to obtain $\sigma_{\text{CD-CT}}$ and $\varepsilon_{\text{CD-CT}}$, then nonane (CT–CM–CT) to get $\sigma_{\text{CD-CM}}$ and $\varepsilon_{\text{CD-CM}}$, heptane (CT2–CM–CT2) to infer $\sigma_{\text{CD-CT2}}$ and $\varepsilon_{\text{CD-CT2}}$, and finally octane (CT2–CM2–CM2–CT2) to get optimal $\sigma_{\text{CD-CM2}}$ and $\varepsilon_{\text{CD-CM2}}$ parameters; B) Optimal σ and ε values for CD:CT

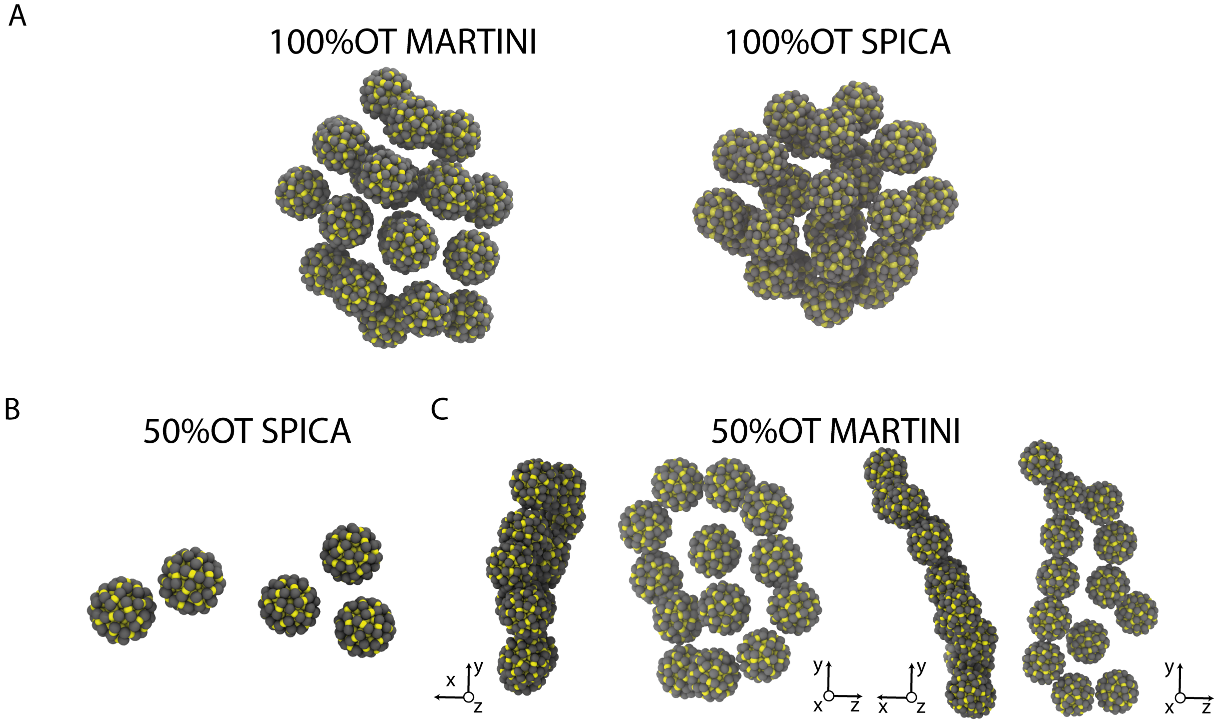

Figure S2: Aggregation behavior of 100%OT and 50%OT SPICA and MARTINI NPs: A) compact aggregates found in 100%OT SPICA and MARTINI; B) prototypical clusters (dimers and trimers) observed in 50%OT SPICA; C) characteristic aggregates detected in 50%OT MARTINI.

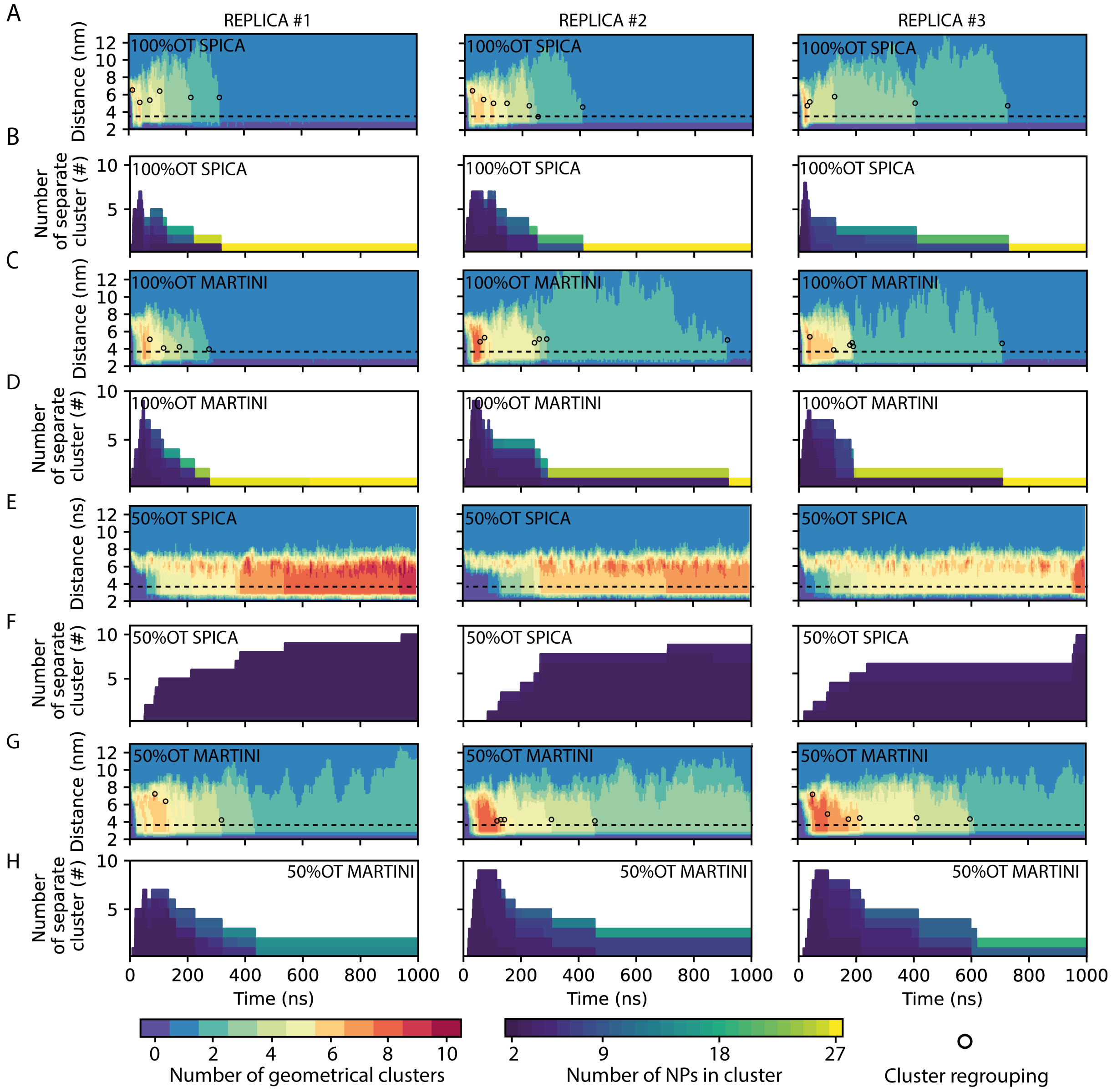

Figure S3: Number of geometrical cluster and number of NPs in each cluster as a function of time. A,C,E,G) number of geometrical cluster as a function of time and distance. B,D,F,H) number of NPs in each cluster. The empty circles highlight the clusters regrouping.

Table S1: Bond parameters for the AuNP: S-CM2 and CM-ASP

Pair	Bond (Å)	K_{b} (kJ mol^{-1})
S-CM2	2.228	77.0
CM-ASP	3.592	9.0

Table S2: Angle parameters for the AuNP: S-CM2-CM2 and CM-CM-ASP

Pair	Angle (Deg)	K_θ (kJ mol^{-1})
S-CM2-CM2	126.00	1.1
CM-CM-ASP	146.95	1.1

Table S3: CD σ and ε non-diagonal parameters optimization: CD:CT

Pair	ε (kJ mol^{-1})	σ (Å)
CD:CT	0.2517	4.0688
