# Supplementary material for: Development of a coarse-grained model for surface-functionalized gold nanoparticles: towards an accurate description of their aggregation behavior
Source: Soft Matter. 2023 Apr 17;19(18):3290–300. doi: 10.1039/d3sm00094j (PMC10170483; doi:10.1039/d3sm00094j)
Supplement: SM-019-D3SM00094J-s002 [file SM-019-D3SM00094J-s002.zip › Royal_Society_of_Chemistry_template_for_preparing_your_submission_to_Soft_Matter_using_Overleaf.pdf]

Soft Matter

ARTICLE TYPE

Cite this: DOI: 00.0000/xxxxxxxxxx

This is the title†

Full Name,*[a] Full Name,[b‡] and Full Name[a]

Received Date
Accepted Date

DOI: 00.0000/xxxxxxxxxx

The abstract should be a single paragraph which summarises the content of the article. Any references in the abstract should be written out in full *e.g.* [Surname *et al.*, *Journal Title*, 2000, **35**, 3523].

The main text of the article[1] should appear here.

0.1 This is the subsection heading style

Section headings can be typeset with and without numbers.[2]

0.1.1 This is the subsubsection style.

These headings should end in a full point.

0.1.1.1 This is the next level heading. For this level please use `\paragraph`. These headings should also end in a full point.

1 Graphics and tables

1.1 Graphics

Graphics should be inserted on the page where they are first mentioned (unless they are equations, which appear in the flow of the text).[3]

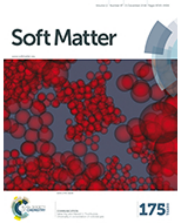

Fig. 1 An example figure caption – the image is from the *Soft Matter* cover gallery.

[a] *Address, Address, Town, Country. Fax: XX XXXX XXXX; Tel: XX XXXX XXXX; E-mail: xxxx@aaa.bbb.ccc*
[b] *Address, Address, Town, Country.*
† Electronic Supplementary Information (ESI) available: [details of any supplementary information available should be included here]. See DOI: 10.1039/cXsm00000x/
‡ Additional footnotes to the title and authors can be included *e.g.* 'Present address:' or 'These authors contributed equally to this work' as above using the symbols: ‡, §, and ¶. Please place the appropriate symbol next to the author's name and include a `\footnotetext` entry in the the correct place in the list.

1.2 Tables

Tables typeset in RSC house style do not include vertical lines. Table footnote symbols are lower-case italic letters and are typeset at the bottom of the table. Table captions do not end in a full point.[4,5]

Table 1 An example of a caption to accompany a table

Header one (units)	Header two	Header three
1	2	3
4	5	6
7	8	9
10	11	12

Adding notes to tables can be complicated. Perhaps the easiest method is to generate these manually.§

2 Equations

Equations can be typeset inline *e.g.* $y = mx + c$ or displayed with and without numbers:

$$A = \pi r^2$$

$$\frac{\gamma}{\varepsilon x} r^2 = 2r \quad (1)$$

You can also put lists into the text. You can have bulleted or numbered lists of almost any kind. The `mhchem` package can also be used so that formulae are easy to input: `\ce{H2SO4}` gives H_2SO_4.

For footnotes in the main text of the article please number the footnotes to avoid duplicate symbols. *e.g.* `\footnote[num]{your text}`. The corresponding author ∗ counts as footnote 1, ESI as footnote 2, *e.g.* if there is no ESI, please start at [num]=[2], if ESI is cited in the title please start at [num]=[3] *etc.* Please also cite the ESI within the main body of the text using †. For the reference section, the style file `rsc.bst` can be used to generate the correct

§ Footnotes should appear here. These might include comments relevant to but not central to the matter under discussion, limited experimental and spectral data, and crystallographic data.

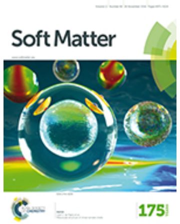

Fig. 2 An image from the *Soft Matter* cover gallery, set as a two-column figure.

Table 2 An example of a caption to accompany a table – table captions do not end in a full point

Header one	Header two	Header three	Header four	Header five	Header six	Header seven
1	2	3	4	5	6	7
8	9	10	11	12	13	14
15	16	17	18	19	20	21

reference style.

3 Conclusions

The conclusions section should come in this section at the end of the article, before the Conflicts of interest statement.

Author Contributions

We strongly encourage authors to include author contributions and recommend using CRediT for standardised contribution descriptions. Please refer to our general author guidelines for more information about authorship.

Conflicts of interest

In accordance with our policy on Conflicts of interest please ensure that a conflicts of interest statement is included in your manuscript here. Please note that this statement is required for all submitted manuscripts. If no conflicts exist, please state that "There are no conflicts to declare".

Acknowledgements

The Acknowledgements come at the end of an article after Conflicts of interest and before the Notes and references.

Notes and references

1 A. Abarca, P. Gómez-Sal, A. Martín, M. Mena, J. M. Poblet and C. Yélamos, *Inorg. Chem.*, 2000, **39**, 642–651.
2 C. D. Abernethy, G. M. Codd, M. D. Spicer and M. K. Taylor, *J. Am. Chem. Soc.*, 2003, **125**, 1128–1129.
3 F. A. Cotton, G. Wilkinson, C. A. Murillio and M. Bochmann, *Advanced Inorganic Chemistry*, Wiley, Chichester, 6th edn, 1999.
4 A. J. Arduengo, III, H. V. R. Dias, R. L. Harlow and M. Kline, *J. Am. Chem. Soc.*, 1992, **114**, 5530–5534.
5 L. N. Appelhans, D. Zuccaccia, A. Kovacevic, A. R. Chianese, J. R. Miecznikowski, A. Macchioni, E. Clot, O. Eisenstein and R. H. Crabtree, *J. Am. Chem. Soc.*, 2005, **127**, 16299–16311.
