# Supplementary material for: Development of a coarse-grained model for surface-functionalized gold nanoparticles: towards an accurate description of their aggregation behavior
Source: Soft Matter. 2023 Apr 17;19(18):3290–300. doi: 10.1039/d3sm00094j (PMC10170483; doi:10.1039/d3sm00094j)
Supplement: SM-019-D3SM00094J-s002 [file SM-019-D3SM00094J-s002.zip › head_foot/RF.pdf]

Journal Name, [year], [**vol.**],
